# Quantitative Electroencephalogram Standardization: A Sex- and Age-Differentiated Normative Database

**DOI:** 10.3389/fnins.2021.766781

**Published:** 2021-12-17

**Authors:** Juhee Ko, Ukeob Park, Daekeun Kim, Seung Wan Kang

**Affiliations:** ^1^iMediSync Inc., Seoul, South Korea; ^2^National Standard Reference Data Center for Korean EEG, Seoul National University College of Nursing, Seoul, South Korea

**Keywords:** QEEG, standardization, age, sex, normative, database, brain disease

## Abstract

We describe the utility of a standardized index (Z-score) in quantitative EEG (QEEG) capable of when referenced to a resting-state, sex- and age-differentiated QEEG normative database (ISB-NormDB). Our ISB-NormDB comprises data for 1,289 subjects (553 males, 736 females) ages 4.5 to 81 years that met strict normative data criteria. A de-noising process allowed stratification based on QEEG variability between normal healthy men and women at various age ranges. The ISB-NormDB data set that is stratified by sex provides a unique, highly accurate ISB-NormDB model (ISB-NormDB: ISB-NormDB-Male, ISB-NormDB-Female). To evaluate the trends and accuracy of the ISB-NormDB, we used actual data to compare Z-scores obtained through the ISB-NormDB with those obtained through a traditional QEEG normative database to confirm that basic trends are maintained in most bands and are sensitive to abnormal test data. Finally, we demonstrate the value of our standardized index of QEEG, and highlight it’s capacity to minimize the confounding variables of sex and age in any analysis.

## Introduction

Electroencephalograms (EEGs) measure electrical activity in the brain and can detect functional abnormalities in the form of abnormal brain waves and signal components. Quantitative EEGs are an advanced digital form of EEGs that make possible more fine-grained, user-independent, complex, and subtle analyses for the differential diagnosis and grading of functional abnormalities.

A growing literature has documented the role of QEEG in novel discoveries and biomarker development for brain diseases including Dementia (Livinţ Popa et al., 2020), Parkinson’s disease (He et al., 2016; Baik et al., 2021), acute ischemic stroke (Bentes et al., 2018), epilepsy (Tedrus et al., 2018), and in clinical psychological syndromes such as ADHD (Arns et al., 2012; Snyder et al., 2015; Angelidis et al., 2016), depression (Kaiser et al., 2018; Arikan et al., 2019), and anxiety (Pavlenko et al., 2009). Much of the previous research used resting-state QEEG to search for novel biomarkers (Angelidis et al., 2016) for the diagnosis and treatment of brain-related diseases.

However, these efforts met with limited success due to lack of standardization of the QEEG databases, and to the complexity of analysis prior to the advent of AI-driven QEEG systems. Standardization of QEEG is essential for it to more fully take its place among the established methods for biomarker development. While standardization of a reference set is essential for any normative comparison, and it is especially so in the case of QEEG, which varies widely from individual to individual. Many factors affect QEEG, such as age, sex, measurement method, and measurement device. All these variables must be carefully considered and controlled for in QEEG, a precaution that was under-appreciated in the first-generation of QEEG research and database development.

The QEEG patterns of normal subjects, carefully constructed into a QEEG normative database, must also include a high N (number of subjects) to fully empower QEEG analysis. Only then can one rely upon the accuracy of parameters such as predicted mean and predicted standard deviation. A properly designed QEEG standardized index can quantify an individual subject’s unique characteristics relative to normal QEEG values, which can be represented by the Z-score, a familiar statistical measure in normal distributions.

Several recent studies (Keizer, 2019; Valdes-Sosa et al., 2021) have described the use of a QEEG normative database. However, these studies describe QEEG normative databases that only account for effects of the age variable on the QEEG distribution. The sex variable has not been considered or controlled in building these traditional QEEG normative databases despite recent studies confirming sex-dependent variation in EEG patterns (Matthis et al., 1980; Karlsgodt et al., 2015; Tomescu et al., 2018; Gartstein et al., 2019), some of which affect the interpretation of pathological findings, thus obscuring details that might be clearly revealed in a sex-differentiated QEEG database (Arns et al., 2016; Rice et al., 2019).

Here, we discuss these studies and highlight the crucial role of the sex variable in the utility of our analyses. We further highlight the value of building each QEEG normative database (ISB-NormDB: ISB-NormDB-Male, ISB-NormDB-Female) with sensitivity to sex, and demonstrate the value of a well-constructed QEEG reference point extracted from the QEEG normative database that demonstrates the value of controlling for sex.

We also propose a modeling process for the resting state QEEG normative database and present validation results for the database that controls for sex and age. Finally, we discuss the utility of our QEEG standardized index obtained by the more sensitive ISB-NormDB.

## Materials and Methods

**FIGURE 1:**
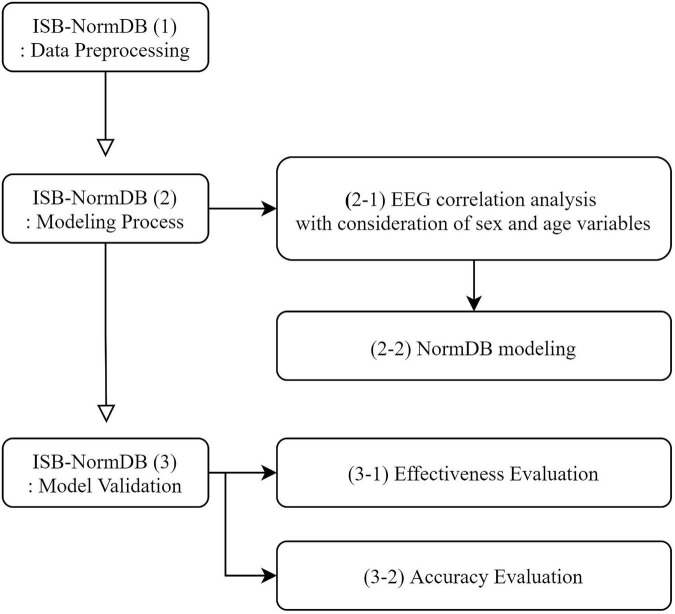
Flow chart for development of the ISB-NormDB.

The ISB-NormDB was systematically developed according to the process outlined in Figure 1.

### ISB-NormDB: Data Preprocessing

#### Participants

To build the ISB-NormDB, EEG data from 1797 human subjects were collected between 2014 and 2019 at the Korean EEG Center at Seoul National University. All procedures were approved by the Research Ethics Committee of the Seoul National University Hospital and informed consent was obtained from each participant or their guardian prior to the study (IRB number: 1801/002-006, 1711/003-004).

**FIGURE 2:**
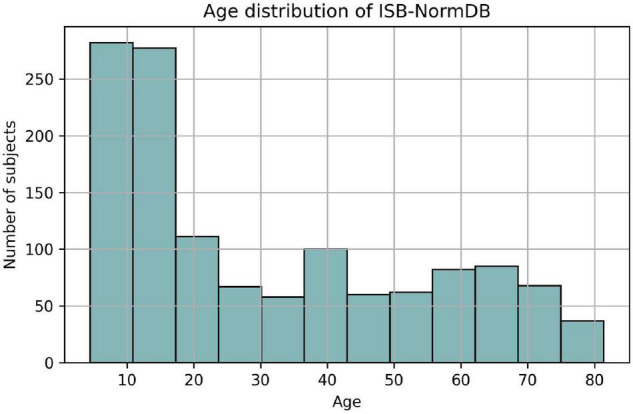
Age distribution of ISB-NormDB.

Subject ages ranged from 4.5 to 81 years, which included a wide distribution from infants to the elderly. The final ISB-NormDB resulted from a strict screening process that yielded a total of 1289 healthy subjects for inclusion in the database. The age distribution of the healthy subjects is as follows (Figure 2).

The rejected subjects were excluded to minimize the influence of confounding variables other than age and sex, and to increase the influence of well-characterized EEG variables on any analyses.

For the strict determination of healthy individuals, four evaluation criteria were established, which included several pre-screening factors, as well as cognitive, emotional, and behavioral factors.

In the pre-screening stage, each subject was evaluated to exclude those with any history of psychiatric or neurological disease, and or a history of problematic academic or social activities. Further, subjects were excluded if there was a history of head trauma or epilepsy, significant behavioral or conduct disorders, or of medical treatment that may have reasonably affected brain function.

In the evaluation stage for cognitive function, the assessments and exclusion criteria differed across four age groupings: infants (4–6 years), children (7–19 years), Adult 1 (20–49 years), and Adult 2 (≥ 50 years).

Wechsler Preschool and Primary Scale of Intelligence (K-WPPSI), CNS Vital Signs (CNSVS), and Mini-Mental State Exam (MMSE) were used to measure cognitive function.

The K-WPPSI is a cognitive function test for preschoolers aged 4 to 6 years. The major tests include block Design (block), maze, picture concepts (picture), vocabulary, and similarities tests. The percentile score is obtained using the raw score (mean = 100, standard deviation = 15).

The evaluation criteria for the raw score of each test are as follows.: 70 or less (percentile score < 2%) is “Extremely Low,” 70–79 is “Borderline” (2% ≤ percentile score < 8%), 80–89 is “Low Average”, 90–109 is “Average”, 110–119 is “High Average”, 120–129 is “Superior”, and 130+ is “Very Superior.”

The CNSVS is a neurocognitive test for children aged ≥7 years. It mainly consists of symbol digit coding, reasoning, verbal memory, and visual memory tests. The evaluation criteria for the percentile score of each test are as follows: “Above Average” (raw score ≥ 109) for 74% or more, “Average” for 25–74% (90≤raw score < 109), and “Low Average” for 9–24% (80≤raw score < 90), 2–8% is “Low” (70≤raw score < 80), and less than 2% is “Very Low” (raw score < 70).

The MMSE test measures cognitive impairment in adults aged ≥ 50 years. Scores at 24–30 are classified as “Normal,” scores at 20–23 are “Mild Dementia,” scores at 10-19 are “Moderate Dementia,” and scores at 9 are “Severe Dementia.” A raw score with a percentile score of 7% is 25.

Table 1 presents the function criteria used to establish the cognitive health of subjects for each age group.

**TABLE 1:** Criteria for inclusion based on cognitive function.

Age group	Infants (4–6 y)		Children and adolescents (7–19 y)		Adult 1 (20–49 y)		Adult 2 (≥ 50 y)	
Cognitive function test	K-WPPSI	Block	CNSVS	Symbol Digit coding	CNSVS	Symbol Digit coding	CNSVS	Symbol Digit coding
		Maze		Reasoning		Reasoning		Reasoning
		Picture		Shift Attention Test		Shift Attention Test		Shift Attention Test
		Vocabulary		Verbal Memory IR and DR		Verbal Memory IR and DR		Verbal Memory IR and DR
		Similarity		Visual Memory IR and DR		Visual Memory IR and DR		Visual Memory IR and DR
		Digit Span						MMSE
Exclusion criteria	K-WPPSI and Digit Span one out of six		CNSVS one out of five tests percentile < 2% or				CNSVS and MMSE one out of six	
	tests percentile < 2% or three tests < 7%							
			CNSVS three out of five tests percentile < 7%				tests percentile < 2% or three tests < 7%	

Child Behavior Check List (K-CBCL), State Trait Anxiety Inventory (STAI-KYZ), Child Depression Inventory (CDI), and Beck Depression Inventory (BDI) were used as evaluation tests for emotional function.

The K-CBCL 1.5–5 test identifies problematic behaviors in children aged 18 months to 5 years. The main evaluations include depression and anxiety. The evaluation criteria for the percentile scores of each test are as follows: More than 98% falls under “Clinical Range,” 93–98% represents “Borderline Clinical Range,” and less than 93% is considered as the “Normal range.”

The STAI-KYZ is a test to measure emotional state and anxiety levels in children aged ≥ 6 years. The test measures state anxiety and trait anxiety. The classification criteria for determining the “Risk Group” and “High-risk Group” differ according to age and sex. The highest score is 80, and the classification criteria are as shown in Table 2 below.

**TABLE 2:** Classification criteria of STAI-KYZ.

Test name	Age group	7 ≤ age < 20	20 ≤ age < 30	Age ≥ 30
State anxiety	risk group (male/female)	59–63/62–66	58–62/62–66	56–60/56–60
	high-risk group (male/female)	64–/67–	63–/67–	61–/61–
Trait anxiety	risk group (male/female)	63–67/65–69	58–62/62–66	60–64/60–64
	high-risk group (male/female)	68–/70–	63–/67–	65–/65–

The CDI measures the degree of depression in children aged ≥ 6 years. The BDI measures that in adults aged ≥ 20 years. For CDI, the highest score is 54, and scores below 21 are classified as “Normal,” those with scores of 22 to 25 are classified as “Mild,” those with scores of 26 to 28 are classified as “Risk,” and those with scores of 29 or higher are classified as “High-risk.” For BDI, the highest score is 63. A score of ≤ 9 is classified as “Normal,” 10-15 is classified as “Mild,” 16–23 is classified as “Risk,” and ≥ 24 is classified as “High-risk.”

Table 3 presents the emotional function criteria used to establish the emotional health of subjects in each age group.

**TABLE 3:** Criteria for inclusion based on emotional function.

Age group	Infants (4–6 y)		Children and adolescents (7–19 y)		Adult (20–81 y)	
Emotional evaluation test	K-CBCL 1.5-5	Depression, anxiety	STAI-KYZ	State anxiety	STAI-KYZ	State anxiety
				Trait anxiety		Trait anxiety
			K-CDI	Depression	BDI	Depression
Exclusion criteria	Depression, anxiety percentile > 98%		One of the three subdomains is a high-risk group,			
			or two or more at-risk groups.			

For the behavioral evaluation, the K-CBCL 1.5-5 for infants and the K-CBCL 6-18 for children and adolescents were used. The behavioral evaluations comprised direct observation by a clinician or a report by a guardian. Table 4 presents the behavioral function criteria used to establish the behavioral health of the infant and child/adolescent groups.

**TABLE 4:** Criteria for inclusion based on behavioral function.

Age group	Infants (4– 6 y)	Children and adolescents (7–19 y)
Behavior evaluation	K-CBCL 1.5-5	K-CBCL 6-18
Exclusion criteria	Expert evaluation	Expert evaluation

The evaluation and screening process identified 553 males and 736 females, comprising 1,289 subjects that passed the strict health criteria.

#### Electroencephalogram Data Measurement and Denoising Procedures

Electroencephalogram (EEG) was measured in 19-channels (Fp1, Fp2, F7, F3, Fz, F4, F8, T3, C3, Cz, Ñ4, T4, T5, P3, Pz, P4, T6, O1, O2) from subjects’ scalps at sites corresponding to the international 10-20 system. The measurement consisted of 4 min with eyes closed, and another 4 min with eyes open, all in a resting state.

Electroencephalogram (EEG) preprocessing was performed to denoise all data and minimize the effects of artifacts. During the first stage of EEG preprocessing, the signals were sampled at 250 Hz and filtered with a bandpass filter of 1∼45.5 Hz range. The EEG were then passed through a notch filter in preparation for downstream processing, including re-referencing (CAR), bad epoch rejection (ASR), and advanced mixture independent component analysis (amICA). Finally, artifacts identified via electromyogram (EMG) and electrooculogram (EOG) were removed to yield cleaned QEEG normative data. All EEG preprocessing processes, sensor-level data, source-level data calculation and extraction were performed using a cloud-based AI-driven auto-analyzing platform (iSyncBrain™, iMediSync, Inc.^1^).

### ISB-NormDB: Modeling Process

#### Electroencephalogram Correlation Analysis With Consideration of Sex and Age Variables

To identify variations between males and females in EEG features with respect to age, two experiments were conducted for two different age groups: a young group (15 < age < 20) and an adult group (20 < age < 40), using the source-level data-theta power band (4∼8 Hz). The theta wave was targeted because it has been widely investigated as a biomarker for disease states related to cognitive and memory performance. Previous studies (Klimesch, 1999; Angelidis et al., 2016; Baik et al., 2021) have indicated that this band is uniquely sensitive to abnormal changes in the brain.

In the first experiment, a total of 170 subjects (85 males, 85 females) from the young group were used; in the second experiment, a total of 176 subjects (88 males, 88 females) from the adult group were used.

For the sex difference test, we applied Student’s *t*-test in cases where the source-level features passed both the Shapiro-Wilk normality test and the Levene’s equality of variance test. Otherwise, Welch’s *T*-test was performed. A nonparametric Mann–Whitney *U* test was performed for features that did not satisfy normality.

#### NormDB Modeling

##### Previous Modeling Process

When the distribution of QEEG data was checked before modeling, it was clearly skewed to the left where skewness was over 1. Since this would be considered a severely biased distribution, it would be impossible to obtain the optimal predicted mean when modeling in this distribution. To solve this problem, log transformation of QEEG data features, involving sensor-level and source-level data, was performed to alleviate the bias observed when combining ages, which made it possible to obtain the optimal predicted mean.

##### Modeling Methods

After log transformation to address the distribution bias in the QEEG features, the resulting predicted mean values were curve-fitted using several different methods. After several trials, the best modeling method was chosen. The discarded QEEG normative database modeling methods divided the QEEG data distributions by age band, wherein the mean and standard deviation are calculated to obtain a standardized score, in this case a Z-score, to correct the effect of age.

However, this sort of age band method risks disconnection problems between successive age bands. For example, if the age of 50 is at the boundary between bands, the difference between 49.9 and 50.1 is only 0.2 years. However, since these two numbers belong to different bands, there may be cases where a mean and standard deviation with a large difference must be used.

For this reason, the curve fitting method, which is a continuous method, was selected.

There are three main methods for constructing a curve fitting model: linear regression, nonlinear regression, and black box machine learning (neural network). Among these three, we selected nonlinear regression – generalized additive models (GAM) using the spline method (Hastie and Tibshirani, 1986). The nonlinear regression method is an intermediate model that combines the advantages of linear regression with those of black box machine learning (neural network).

Linear regression is a method in which variables are linearly connected and produce predicted values. While this has some advantages in interpreting the model, it also has disadvantages due to the simplicity of the model which only allows good fitting on data with simple tendencies. This increases the possibility of underfitting EEG data having complex tendencies. Likewise, while a black-box machine learning (neural network) model may have good prediction capability for data with complex nonlinear tendencies, it may be insufficient for analyzing and inferring the process behind the model.

Nonlinear regression (GAM), which combines the strengths of these two models, can fit more complex nonlinear relationships than linear regression because it uses the spline method. In addition, statistical inference allows us to better understand and explain the structure of the model.

### ISB-NormDB: Model Validation

#### Effectiveness Evaluation

To compare trends between the two QEEG normative databases, 148 subjects aged 5 to 90 years (eyes-closed data) and 96 subjects aged 5 to 90 years (eyes-open data) were randomly selected. Their EEG data were input to each QEEG standard database and Z-scores were extracted. A correlation analysis was then performed on the z-scores extracted from each QEEG normative database.

The Z-score is a standardized score that minimizes the influence of variables affecting the EEG in each QEEG normative database, which follows a standard normal distribution with a mean of 0, a variance of 1, and is denoted as Z∼N (0,1).

A main benefit of z-score analysis is that it permits comparison between databases that have marked differences in modeling methods and data compositions. When z-scores of two QEEG normative databases are compared on same standard normal distribution, a trend comparison can be performed. Moreover, a high correlation between database Z-scores suggests that two databases capture the same basic normal-state trends, in spite of differences in data composition, measurement method, and de-noising method.

To compare trends with our ISB-NormDB database, we selected the “qEEG-Pro” database, an FDA-approved commercial database. Table 5 summarizes the characteristics of the two databases.

**TABLE 5:** Characteristics of the ISB-NormDB database and the qEEG-Pro database.

	ISB-NormDB		qEEG-Pro	
	Eyes closed (*n* = 1289)	Eyes open (*n* = 1290)	Eyes closed (*n* = 1232)	Eyes open (*n* = 1482)
Age range	4.5 ∼ 81		6 ∼ 83	
Data collection period	2012∼2019		2004 ∼ 2013	
Montage	Average reference		Linked ear reference	
Age regression method	Nonlinear regression (GAM)		Sliding window	

#### Accuracy Evaluation

To evaluate the accuracy of the ISB-NormDB (ISB-NormDB-Male, ISB-NormDB-Female) in controlling for the effects of sex and age, the Z-score of ISB-NormDB-Total (ISB-NormDB-Male+Female), which controls age variables without discriminating by sex, was compared with the Z-score of ISB-NormDB.

We analyzed data on a male with developmental disabilities (aged 10.2 years), a male diagnosed with amnestic mild cognitive impairment (aMCI) (aged 78 years), and a female with anxiety (aged 43 years) excluded from the ISB-NormDB.

First, zscore1 was extracted by inputting it into the ISB-NormDB (ISB-NormDB-Male or ISB-NormDB-Female) suitable for the sex of each subject, and zscore2 was extracted by inputting it into the ISB-NormDB-Total. The two Z-scores were then compared.

Source-level data–theta band power was used as the test feature of a male with developmental disorder and a male diagnosed with aMCI, and Source-level data–beta3 was used as the test feature of a female with anxiety. The theta and beta3 bands were used because the theta band is known to be sensitive to developmental disorders and aMCI, and the beta3 band is known to be sensitive to anxiety.

Subjects suffering anxiety exhibit greater beta3 power than do normals (Ribas et al., 2018; Tarrant et al., 2018).

Likewise, children with developmental disorders exhibit greater theta power than do normal children (Snyder and Hall, 2006; Arns et al., 2008; Robertson et al., 2019). There is also a tendency toward greater theta power in those with aMCI compared with normals (Wolf et al., 2003; Roh et al., 2011; Musaeus et al., 2018). Notably, the increased theta power also entails slowing down of the wave.

## Results

### Correlation Analysis With Consideration of Sex and Age Variables

**FIGURE 3:**
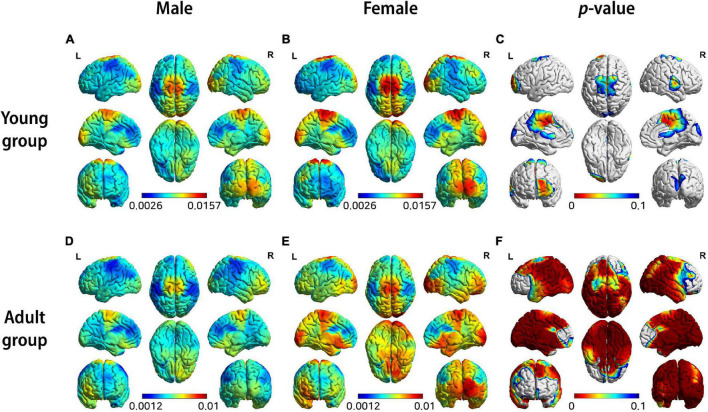
3D brain images of both female and male groups showing median source-level theta band power by color: **(A)** males in young group, **(B)** females in young group, **(D)** males in adult group, **(E)** females in adult group, **(C,F)** Visualization of statistical differences by *T*-test highlighting zones having p-values ≤ 0.1.

Figure 3 shows the results of each sex difference test for source-level theta band power by separating the young group (15 < age < 20) from the adult group (20 < age < 40). This result shows a sex differences as a function of age.

Two key insights were provided by these two experiments. First, it can be seen that the brain areas showing significant differences between males and females differ markedly between the young group and the adult group (Figures 3C,F). Second, the results of each experiment with the young and adult groups reveal that brain areas show differences of varying significance levels between males and females within each group (Figures 3A,B,D,E). This suggests that, although the sex differences are almost negligible when considered across the entire age range, they become evident when the age groups are separated into smaller ranges wherein significant differences are evident between the two sex groups.

Thus, the importance of sex as a variable affecting interpretations of QEEG becomes apparent, and this underscores the importance of building a sex-discriminated normative database. We suggest that a well-designed QEEG normative database must be capable of discriminating both age and sex.

### NormDB Modeling

In the present study, QEEG normative database models (GAM models), which minimizes the effects of sex and age, were built for each band: delta (1∼4 Hz), theta (4∼8 Hz), alpha (8∼12 Hz), and beta (12∼30 Hz).

**FIGURE 4:**
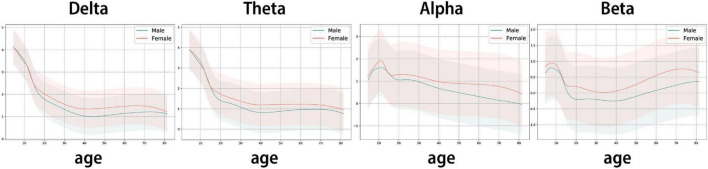
Trend line of GAM curve fitting model: The estimated log-transformed sensor-level feature as age changes. The interval is the 95% prediction interval of the GAM model. Y axis: ln (Sensor-level feature – band power) (band = delta, theta, alpha, beta), X axis: age. Red line: estimated mean line for females, blue line: estimated mean line for males.

Three statistical trends were observed in Figure 4:

1. Delta and theta waves, which are slow waves, show a sharp decline in infants, children, and adolescents (ages 4.5 to 19 years), followed by relatively stable trends after the age of 20.
2. Alpha waves fluctuate in infants, children, and adolescents (4.5∼19 years), followed by a steady decrease after the age of 20.
3. Beta waves decrease in infants, children, and adolescents (4.5∼19 years), followed by a steady increase after the age of 20.

The rapid EEG changes in children and adolescents, the relative stability of EEG characteristics in adulthood, and the decrease in delta, theta, and alpha, combined with the increase in beta with age, are patterns that have been well established in previous studies (Smit et al., 2011; Barry and De Blasio, 2017). Thus, the present model is consistent with the prior research.

Therefore, the predicted mean EEG and standard deviation EEG corresponding to all consecutive ages can be obtained with the estimated mean and prediction interval of the ISB-NormDB model. Further, by calculating the Z-score with the mean and standard deviation obtained, a QEEG standardized index can be extracted.

### Model Validation

#### Effectiveness Evaluation

To evaluate the effectiveness of the ISB-NormDB database, the results of examining the correlation of Z-scores of each band of the same data with the FDA approved database qEEG-Pro are as follows.

**FIGURE 5:**
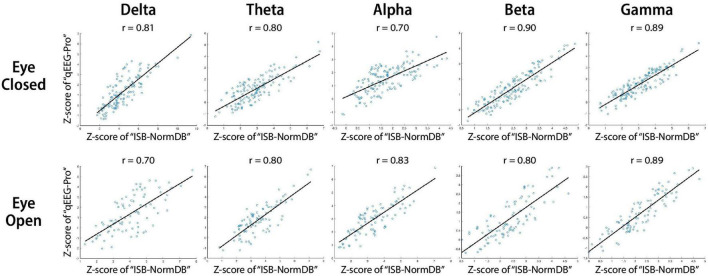
Scatter plots and least squares lines to assess correlation of Z-scores between the ISB-NormDB and the qEEG-Pro databases.

The Z-score comparison between the two QEEG standard databases, including analysis of all QEEG band features (delta, theta, alpha, beta, gamma) in both the eyes-closed (EC) and eyes-open (EO) conditions revealed a correlation of *r* > 0.7. Thus, the Z-scores of the two QEEG normative databases can be considered to be highly correlated as a whole (Figure 5).

This analysis suggests that the ISB-NormDB database and the qEEG-Pro database share the same basic normal-state trends. Further, these findings suggest that the ISB-NormDB can be considered to be a cross-validated QEEG normative database across a wide range of ages.

#### Accuracy Evaluation

To evaluate the capacity of the ISB-NormDB to minimize the effects of sex and age, we compared the ISB-NormDB-Total Z-score and the ISB-NormDB (ISB-NormDB-Male or ISB-NormDB-Female) Z-score for abnormal data, as follows.

**FIGURE 6:**
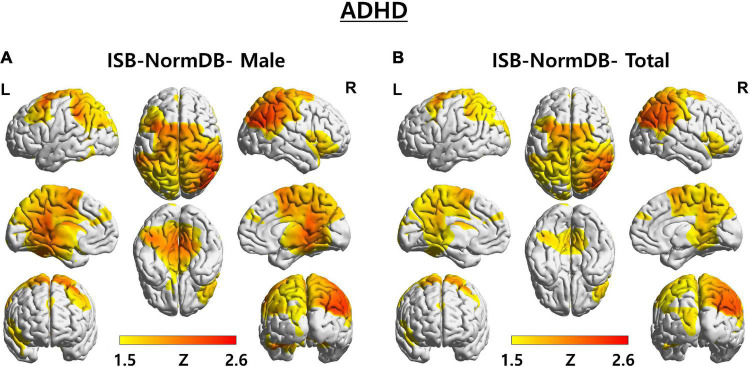
3D brain images of both ISB-NormDB-Male and ISB-NormDB-Total showing Z-score of source-level theta band power of test data with ADHD by color: **(A)** Z-score extracted from ISB-NormDB-Male and **(B)** Z-score extracted from ISB-NormDB-Total.

In one first case, we determined the Z-score of a young male (aged 10.2 years) with developmental disabilities. We compared zscore1 calculated with parameter values (mean, standard deviation) of ISB-NormDB-Male using only male data, with zscore2 calculated with parameter values (mean, standard deviation) of ISB-NormDB-Total using both male and female data. In this comparison, the left middle frontal area and both the posterior cingulate and para-hippocampal areas showed a zscore1 that was higher than zscore2 (Figure 6).

Consistent with previous studies, the left middle frontal area is known to show higher theta power in ADHD relative to normal subjects (Hermens et al., 2005). Likewise, abnormalities in the posterior cingulate area are known symptoms of ADHD (Leech and Sharp, 2013), and the corresponding Z-score changes shown here are consistent with those reports.

**FIGURE 7:**
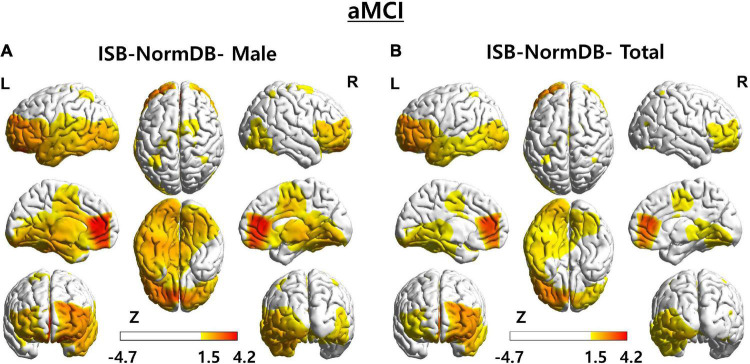
3D brain images of both ISB-NormDB-Male and ISB-NormDB-Total showing Z-score of source-level theta band power of test data with aMCI by color: **(A)** Z-score extracted from ISB-NormDB-Male and **(B)** Z-score extracted from ISB-NormDB-Total.

As a second case, we determined the Z-score of a male (aged 78 years) with aMCI (Figure 7).

These data reveal a typical aMCI pattern, wherein abnormality in the left temporal lobe and in the overall frontal lobe (higher theta band power than normal) (Roh et al., 2011; Bian et al., 2014) is present. As evident in Figure 7, the zscore1 shows temporal lobe and frontal lobe abnormality (Figure 7A) more clearly than does the zscore2 (Figure 7B). So, as a result of comparing the Z-score of the source power-theta band, the zscore1 (Figure 7A) of ISB-NormDB-Male (normative database with minimal influence of sex) could detect a more certain abnormal ROI than the zscore2 (Figure 7B) extracted from ISB-NormDB-Total.

**FIGURE 8:**
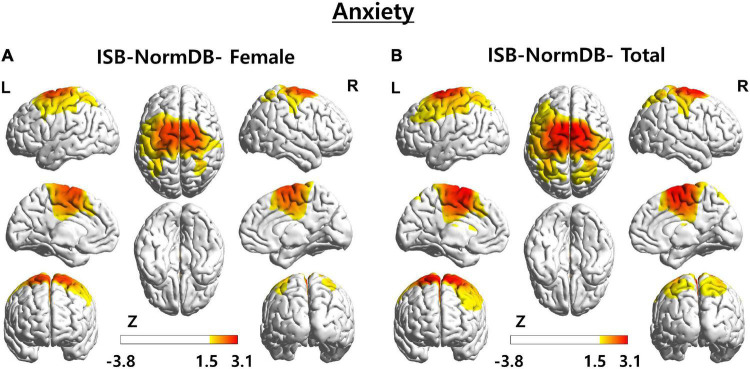
3D brain images of both ISB-NormDB-Female and ISB-NormDB-Total showing Z-score of source-level beta3 band power of test data with anxiety by color: **(A)** Z-score extracted from ISB-NormDB-Female and **(B)** Z-score extracted from ISB-NormDB-Total.

As a third case, we analyzed the Z-score of a female (aged 43 years) with anxiety (Figure 8).

As a result of comparing the Z-score of the source power-beta3 band, zscore2 (Figure 8B) of ISB-NormDB-Total shows an ambiguous ROI as the central region including some portions of the frontal and parietal lobes. On the other hand, it can be seen that zscore1 (Figure 8A) is associated with a specific ROI.

These three test cases offer further support for the claim that a QEEG normative database that considers both sex and age is more sensitive and accurate than one that only considers for one of these two variables.

## Discussion

Previous attempts (Thatcher et al., 2003; Keizer, 2019; Valdes-Sosa et al., 2021) to improve the utility of QEEG normative databases focused on controlling for confounding results attributable to the age variable. We’ve extended this effort to include control of the sex variable as well. We empirically tested the value of controlling for the sex and age variables by comparing Z-scores between QEEG normative databases. Our findings revealed a trend line of the model that is consistent with previous findings (Smit et al., 2011; Barry and De Blasio, 2017), and we found a high correlation between z-scores extracted from two different QEEG normative databases for the same data. The present results comprise a cross-validation of the ISB-NormDB across a wide range of ages. In addition, it was confirmed that anomaly detection performance was improved by detecting abnormal values more sensitively or accurately when the age and sex variables were controlled together as compared to when only age was controlled (Figures 6–8). Thus, the ISB-NormDB, which uniquely minimizes the confounding influences of both age and sex, is capable of extracting a standardized index (Z-score) that is highly sensitive to anomalies, making it highly promising for research, monitoring, and treatment of brain-related diseases affected by age and sex.

### Comparison to Previous Studies

Among the QEEG normative databases, there are several previously developed databases such as EEGPro, Neuroguide, HBI, BrainDx, etc. Comparing the characteristics of these databases with our ISB-NormDB is as follows.

The present QEEG normative database analyses highlight the most essential characteristics of EEG by minimizing the influence of variables other than those of EEG spectra power. Normal people were selected through four strict standards of normality, and were classified according to sex. QEEG normative database modeling was performed according to age using subjects’ EEG data, minimizing the effects of abnormal factors, sex, and age on the QEEG. Further, any anticipated confounds due to this being a Korean-subjects database were addressed by minimizing the influence of race and ethnic factors. Finally, by presenting a standardized distribution of gamma bands that were not considered in previous QEEG normative database studies, the present study represents a significant methodological advance over that in previous studies.

### Application and Potential Contribution of Quantitative Electroencephalogram Standardization Index

The QEEG standardized index(Z-score) can be applied in many medical fields.

#### Application of Diagnosis and the Direction Treatment

First, it can provide quantitative biodata for the diagnosis of various mental disorders identified by DSM-5, the standardized and diagnostic tool for psychiatric disorders (American Psychiatric Association, 2013; Wakefield, 2016). Z-score assessments linked to an improved QEEG normative database can also provide more granular data and localization to support individualized medical treatment in psychiatric and neurodegenerative disorders. For example, diagnostic and treatment choices in ADHD might be guided by Z-score variations across locations, such as frontal versus occipital lobe variations, as opposed to blinded or whole brain treatments.

#### Application of Treatment Using Neurofeedback

A QEEG standardization index (Z-score) is commonly used in EEG neurofeedback by expressing statistically abnormal EEG power and matching it to anatomical locations. Neurofeedback is a training that normalizes brain function by conditioning subjects to suppressor strengthen EEG components in specific regions of the brain. It is capable of addressing disease states through repetitive training that can transform some abnormal neural functioning to normal functioning. Neurofeedback as a clinical intervention has been developed over many years by several pioneering clinicians (Thatcher, 1998; Cantor, 1999). Many previous studies have focused on the effectiveness of Z-score-based neurofeedback for treating a wide range of diseases. In the case of attention deficit/hyperactivity disorder (ADHD) (Medici, 2018), greater improvement in executive function, behavior, and attention were associated with QEEG neurofeedback compared with conventional treatment [Methylphenidate (MPH) treatment]. In the case of traumatic brain injury (TBI), several studies have shown the efficacy of neurofeedback for improving cognition, behavior, and physical dysfunction (Bennett et al., 2017; Gray, 2017; Brown et al., 2019; Gupta et al., 2020; Faridi et al., 2021). There is active ongoing research into expanding the applications of QEEG neurofeedback for the treatment of other psychiatric and brain-related diseases. Further, findings such as those in the present study, should expand the application of QEEG neurofeedback using Z-score analysis derived from age- and sex-differentiated QEEG normative databases.

Finally, this and future AI-driven QEEG indexes will play crucial roles in drug development strategies, patient response monitoring, patient selection, biomarker development, and myriad other applications, especially in conditions where the effects of sex, age, and other variables influence their presentation (Hermens et al., 2005; Tement et al., 2016; Rice et al., 2019).

### Limitation

There are several limitations in this study. In an experiment in which correlation analysis was performed with z-scores obtained from each database for the same EEG data, a high correlation was obtained when compared with the qEEG-Pro database. However, since the z-score of ISB was compared with the z-score of qEEG-Pro, a separate database, additional verification of ISB-NormDB through more comparisons with several other QEEG normative databases would provide wider verification.

It is noteworthy that a previous study (Keizer, 2019) has confirmed a high correlation between Z-scores derived from qEEG-Pro and those of “NeuroGuide,” which is another commercial database. Thus, one may expect a similarly high correlation between “NeuroGuide” and ISB-NormDB using similar analyses, which will be the subject of future research.

## Data Availability Statement

The data that support the findings of this study are available from the corresponding author, SK drdemian@snu.ac.kr and seungwkang@imedisync.com upon reasonable request.

## Ethics Statement

The studies involving human participants were reviewed and approved by Research Ethics Committee of the Seoul National University Hospital. Written informed consent to participate in this study was provided by the participants’ legal guardian/next of kin.

## Author Contributions

All authors listed have made a substantial, direct, and intellectual contribution to the work, and approved it for publication.

## Conflict of Interest

JK, UP, and DK are employed by iMediSync Inc. SK founded iMediSync Inc and now is affiliated in the company.

## Publisher’s Note

All claims expressed in this article are solely those of the authors and do not necessarily represent those of their affiliated organizations, or those of the publisher, the editors and the reviewers. Any product that may be evaluated in this article, or claim that may be made by its manufacturer, is not guaranteed or endorsed by the publisher.

## Footnotes

1
https://isyncbrain.com
